# Protocol for a randomized controlled trial: exercise-priming of CBT for depression (the CBT+ trial)

**DOI:** 10.1186/s13063-024-08495-x

**Published:** 2024-10-07

**Authors:** Jacob D. Meyer, Shania J. E. Kelly, John M. Gidley, Jeni E. Lansing, Seana L. Smith, Sydney L. Churchill, Emily B. K. Thomas, Simon B. Goldberg, Heather C. Abercrombie, Thomas A. Murray, Nathaniel G. Wade

**Affiliations:** 1 Department of Kinesiology, Iowa State University, Ames, IA USA; 2 Department of Kinesiology, University of Wisconsin-Madison, Madison, WI USA; 3 Department of Psychological and Brain Sciences, University of Iowa, Iowa City, IA USA; 4 Department of Counseling Psychology, University of Wisconsin, Madison, WI USA; 5 Center for Healthy Minds, University of Wisconsin, Madison, WI USA; 6 Division of Biostatistics and Health Data Science, University of Minnesota, Minneapolis, MN USA; 7 Department of Psychology, Iowa State University, Ames, IA USA

**Keywords:** Major depressive disorder, Cognitive behavioral therapy, Exercise, Exercise priming, Treatment augmentation, Process mechanisms, Antidepressant mechanisms, Psychosocial treatment, Nature interaction, Behavioral activation

## Abstract

**Background:**

Depression is a leading cause of disability worldwide, and treatments could be more effective. Identifying methods to improve treatment success has the potential to reduce disease burden dramatically. Preparing or “priming” someone to respond more effectively to psychotherapy (e.g., cognitive behavioral therapy [CBT]) by preceding sessions with aerobic exercise, a powerful neurobiological activator, could enhance the success of the subsequently performed therapy. However, the success of this priming approach for increasing engagement of working mechanisms of psychotherapy (e.g., increased working alliance and behavioral activation) has yet to be formally tested.

**Methods:**

The CBT + trial will be a parallel-arm randomized controlled trial that will recruit 40 adult participants with DSM-5 diagnosed depression (verified with clinical interview) via referrals, mass emails, local flyers, and social media posts. Participants will be randomized to an ActiveCBT or CalmCBT condition. The ActiveCBT group will receive an 8-week CBT intervention primed with 30 min of moderate-intensity aerobic exercise (cycling on a stationary bike at a 13 rating of perceived exertion). The CalmCBT group will receive the same 8-week CBT intervention while resting for 30 min before CBT (i.e., cycling vs no cycling is the only difference). The primary outcome measures will be mean working alliance (assessed with the client version of the Working Alliance Inventory—Short Revised) and mean behavioral activation (self-reported Behavioral Activation for Depression Scale) recorded at each of the 8 therapy sessions. Secondary outcomes include evaluation of state anhedonia and serum brain-derived neurotrophic factor before the active/calm conditions, between the condition and therapy, and after the therapy. Additional exploratory analyses will evaluate group differences in algorithm-generated ratings of therapist-participant interactions via the Lyssn platform.

**Discussion:**

The novel approach of priming CBT with moderate-intensity aerobic exercise evaluated in a randomized controlled trial (CBT + trial) has the potential to demonstrate the usefulness of exercise as an augmentation strategy that improves working mechanisms of therapy and overall treatment outcomes for adults with depression.

**Trial registration:**

ClinicalTrials.gov NCT06001346. Registered on August 21, 2023.

## Background and rationale {6a}

Depression is a leading cause of disability worldwide, with 30% of US adults suffering from a major depressive episode in their lifetime and experiencing multiple episodes is common [38]. Traditional stepped treatment approaches for major depressive disorder (MDD) evaluated in the Sequenced Treatment Alternatives to Relieve Depression (STAR*D) randomized controlled trial (RCT) yielded disappointing results, with > 30% of people not remitting after four successive treatments [59]. Research in the intervening years has not demonstrated meaningful improvements in success rates in usual care [19]. While psychotherapy is a standard front-line treatment for depression, only half of patients respond to therapy, including cognitive behavioral therapy (CBT [18]), and many relapse shortly thereafter [45, 70]. Taken together, the high prevalence and significant number of patients who do not respond to current treatments indicate that there is a critical need for novel approaches to improve MDD treatment success.

From numerous, well-designed RCTs, psychotherapy is effective in treating MDD, but currently provides only a modest benefit above care-as-usual and the passage of time. In general, about 6 in 10 post-therapy patients no longer meet MDD criteria, but almost 5 in 10 also achieved this with care-as-usual [18]. Further, of those whose MDD was successfully treated by CBT, 54% relapse within 24 months [45, 70]. Yet, there is high interest in psychotherapy and exercise as they are often indicated by patients as preferred treatments [22, 25, 49]. Additionally, acutely augmenting psychological therapy may be able to provide greater overall benefits for treating mental health problems [54]. Indeed, Nord and colleagues [54] found acute augmentation of psychotherapy (i.e., altering biological or psychological processes immediately before psychological therapy) significantly reduced transdiagnostic mental health concerns (Hedges’ *g* =  − 0.27), suggesting acute augmentation may be a useful approach to increase the efficacy of mental health treatment. Finally, experiencing “sudden gains” (large improvements between two sessions) during treatment also predicts positive treatment outcomes and lower relapse rates [2, 3, 12, 46, 68, 69]. Acute augmentation that creates more effective individual sessions could increase the likelihood of experiencing sudden gains. In sum, current therapy practice could be more effective, and augmenting with non-pharmacological approaches, which is appealing to patients, may lead to more sudden gains and better overall treatment effects.

Consistently, RCTs show that exercise training is efficacious in acute MDD treatment and preventing relapse [11, 15, 43, 44, 62], but difficulty in starting and maintaining an exercise program have limited the use of exercise training as a primary treatment [66]. Promisingly, exercise has powerful acute psychobiological effects, including immediate improvements in psychological states and short-term enhancement of neuroplastic mechanisms [5, 13, 26, 50, 51, 71]. Given this acute enhancement, the period immediately after exercising may be an opportune time in which other treatments could be augmented if applied sequentially. Past research from our team suggests that depressed mood state and state anhedonia [52, 53] and serum BDNF (a marker of neuroplasticity [51]) are improved for at least 50 min following a 30-min bout of moderate exercise, indicating a window in which to deliver back-to-back exercise and therapy. Indeed, our 10-person pilot randomized controlled trial of exercise immediately before therapy compared to a quiet rest comparator (i.e., ActiveCBT vs CalmCBT) demonstrated the feasibility and acceptability of this approach while also demonstrating the plausibility of potentially greater working alliance, behavioral activation, and antidepressant efficacy in the active condition [52, 53]. This combined approach sequencing moderate aerobic exercise immediately before treatment, however, needs to be tested in a larger trial to determine the potential for exercise priming to improve process mechanisms of CBT and eventual overall treatment success.

## Objectives {7}

Thus, the purposes of the CBT + randomized, controlled trial (*n* = 40) are to (1) demonstrate engagement of target CBT mechanisms (self-reported working alliance and behavioral activation) by exercise priming over quiet rest, (2) evaluate the link between psychological (improved anhedonia) and neuroplastic (increased serum BDNF) in response to exercise priming to target CBT mechanisms, and (3) explore the sensitivity of objective machine learning-based markers (collaboration and empathy) from Lyssn to exercise priming of therapy. To objectively assess therapy sessions, machine learning and natural language processing will be employed via the Lyssn platform to code each session’s content. Exercise priming has high potential translatability as exercise is accessible with no/low-cost (easily added in outpatient treatment centers, medical centers have exercise equipment, can be performed and overseen virtually, etc.). If successful, exercise priming has the potential to improve treatment success and lower relapse rates, significantly reducing the substantial burden of MDD.

## Trial design {8}

This is a 1:1 parallel, randomized controlled trial consisting of 8 weekly visits of CBT, primed by either 30 min of moderate-intensity exercise (*n* = 20; ActiveCBT) or 30 min of quiet rest (*n* = 20, CalmCBT). Thirty-minute segments of a nature documentary series will be shown during the priming periods to standardize the psychological activity performed across the active and calm conditions. Hypotheses are average (1) self-reported working alliance and behavioral activation will be greater in ActiveCBT vs CalmCBT, (2) state anhedonia and BDNF improvements after exercise will be associated with each session’s alliance and activation, and (3) there will be higher objective ratings (e.g., collaboration, empathy) in ActiveCBT sessions than in CalmCBT sessions.

This clinical trial is registered with ClinicalTrials.gov (ClinicalTrials.gov ID: NCT06001346; Protocol ID: 1R61MH129407; registered on 9/23/2023) and funded by the National Institute of Mental Health (NIMH; FAIN#: 5R61MH129407), titled “ActiveCBT for depression: Transforming treatment through exercise priming,” with “CBT + Study” used for public engagement. The Iowa State University Institutional Review Board (IRB) has reviewed and approved all procedures (Study ID: 23–026-00, approval date: 2/21/2023). All items included in the WHO Trial Registration Data Set can be found on ClinicalTrails.gov and herein.

## Methods: participants, interventions, and outcomes

### Study setting {9}

All study procedures are planned at Iowa State University, with data collection and study visits occurring at the College of Human Sciences Research Park Testing Center in Ames, IA, USA, with enrollment beginning September 2023. Potential participants will provide informed consent and be screened for eligibility criteria during an intake visit. Participants will set up their initial therapy session within the next week and continue coming weekly to therapy sessions for 8 visits, followed by a post-therapy visit (within 1 week of their 8th CBT session) and follow-up visits occurring at 20 and 52 total weeks from enrollment to determine potential longer-term effects. Scheduling flexibility (e.g., for vacations, illness, personal reasons) will be included to allow the 8 sessions to occur over 12 weeks with no visits occurring less than 3 days apart.

### Participants, eligibility, and consent {10, 26a, 26b}

The inclusion and exclusion criteria are designed to enroll adults with a primary clinical concern of depression who would be good candidates for beginning individual outpatient therapy. Inclusion criteria for this trial are (1) diagnosed with DSM-5 MDD, confirmed via Structured Clinical Interview for DSM-5 (SCID); (2) current depressive symptoms of at least mild severity defined by a clinician-rated Hamilton Rating Scale for Depression-17 (GRID-HAMD [30] rating ≥ 8; (3 between the ages of 18 and 65 years; (4 either not engaging in any mental health treatment (e.g., medication, psychological, or behavioral or on a stable mental health treatment regimen (of at least 8 weeks and willing to maintain it for the length of the intervention; (5 willing and safe to perform exercise based on the verbally administered Physical Activity Readiness Questionnaire or provides physician approval for study participation; and (6 CBT-naïve within the past 5 years. The CBT-naïve criterion will be systematically assessed during the clinical interview by inquiring about previous counseling or psychotherapy and discussing the therapy approach used in detail (e.g., topics discussed, type of homework to determine if the participant engaged in structured CBT.

Exclusion criteria for this trial are (1) currently pregnant, nursing, or planning to become pregnant, assessed via self-report; (2) severe obesity (BMI ≥ 40) confirmed at the initial visit; (3) diagnosed with lifetime or current psychosis, mania, or bipolar disorder, confirmed via the SCID; (4) diagnosed with current substance use disorder, confirmed via the SCID; (5) pose an imminent risk of self-harm or harm to others (i.e., clinical interviewer recording a “5—Active Suicidal Ideation with Specific Plan and Intent” on the Columbia Suicide Severity Rating Scale (C-SSRS)); and (6) exhibit behavioral disturbance (e.g., aggression, mild-moderate cognitive impairment) that would significantly interfere with study participation, as assessed by clinical research personnel.

A senior researcher will lead the informed consent process during the intake visit. Participants will be asked to read an informed consent form. They will be given a verbal overview of the document and a tour of the research facilities with a chance to talk through each space (condition room, therapy room, phlebotomy room, etc.). When all questions have been answered and if participants decide to enroll, they will sign the informed consent form, completed electronically directly on a secure online data collection platform (REDCap). The research staff will then counter-sign, verifying they witnessed the informed consent process and that the participant consented to participate in the study.

The informed consent document includes language regarding the potential use of data beyond the specific plans of this study. In any and all future analyses, only deidentified data will be used and may be shared for publishing or other repository requirements. If this happens, the data may become publicly available and could be used by researchers for many types of studies or topics (e.g., in the NIMH Data Archive). However, video and audio recordings and transcriptions from the clinical interview or therapy sessions will never be shared. Participants will be able to indicate if they do not want their deidentified data shared in the NIMH Data Archive and will still be able to be enrolled in the trial.

### Interventions and explanation of choice of comparator condition {6b, 11a}

To determine the influence of exercise-priming on cognitive behavioral therapy (CBT) mechanisms, this study employs a time- and attention-matched control using a standard nature documentary series, with identical conditions, aside from the pre-therapy condition (i.e., “ActiveCBT” vs “CalmCBT”). That is, participants randomized to the CalmCBT group will view the same standardized nature videos and complete identical procedures to those randomized to the ActiveCBT group, but while resting quietly for 30 min. The nature video viewing provides a time- and attention-matched control, making pre-therapy conditions identical apart from the exercise performed by participants in the ActiveCBT group.

All participants will complete eight weekly visits. Participants will arrive 50 min prior to the start of each CBT session to complete acute measures and the pre-therapy condition. Participants will begin with questionnaires before completing a 30-min pre-therapy condition (each described below). Questionnaires will be administered again immediately post-condition. Following, participants will be escorted to meet with a trained therapist and begin their individual CBT session in a private room approximately 10 min after the end of the active or calm condition. During sessions 1, 4, and 8, a blood sample will also be collected pre-condition, post-condition, and post-therapy after completing questionnaires at each time point.

#### Active condition (ActiveCBT)

Individuals in the ActiveCBT group will complete 30 min of cycling on a stationary bicycle (Corival Recumbent, Lode, Groningen, The Netherlands) while watching the nature video. These supervised aerobic exercise sessions will consist of steady-state exercise at a moderate intensity corresponding to a “13” or “somewhat hard” rating of perceived exertion (RPE) [10], as has been done by our team in the past [52, 53]. Participants will begin with a 3-min warmup, 24 min of cycling at a moderate intensity, followed by a 3-min cooldown. Monitoring of heart rate (beats per minute), resistance/load (watts), cadence (revolutions per minute), affect (Feeling Scale; [32]), and RPE [10] will occur every minute during the warmup/cooldown and every 5 min throughout the exercise to ensure participant safety and to maintain a moderate intensity. Thirty minutes of moderate exercise was chosen as previous data shows that this prescription improves state anhedonia [52, 53] and increases BDNF in adults with MDD [51] and is perceived as feasible and acceptable pre-therapy [52, 53].

#### Calm condition (CalmCBT)

Participants randomized to the CalmCBT group will view the exact same standardized nature videos as the ActiveCBT group while sitting and resting quietly for 30 min in the same room used for the active condition. Similar to the ActiveCBT procedures, heart rate, affect, and RPE will be monitored every 5 min during the condition.

#### Nature videos

During each condition, participants will watch a standard 30-min segment from a nature documentary to aid in creating a relatively emotionally neutral yet engaging psychological experience that is standardized across conditions. Blue Planet II, Season 1 [7] was selected as the neutral video stimulus because it was available through the duration of the study and contained different video segments for all eight intervention visits. This video depicts similar content to other nature documentaries that have resulted in minimal fluctuations in emotion [36]. The team piloted these videos to assess arousal and overall emotional neutrality. Individuals on the study team watched all episode segments and subsequently completed the video survey (described below). Piloting was completed in two cohorts, one including the study team, which resulted in the removal of episodes 1 and 7 due to high emotional responses and episode 8 as it showed human-nature interactions which may elicit a change in a viewer’s emotional state [9]. Episodes 2–6 were then piloted and rated again, using the same system but with non-senior research staff. After these ratings, episode 6 was removed due to higher emotional response and a lack of neutrality. Final video segments that will be played are as follows: CBT visit 1—episode 2 (minutes 0–30), CBT visit 2—episode 2 (minutes 20–50), CBT visit—episode 3 (minutes 0–30), CBT visit 4—episode 3 (minutes 20–50), CBT visit 5—episode 4 (minutes 0–30), CBT visit 6—episode 4 (minutes 20–50), CBT visit 7—episode 5 (minutes 0–30), and CBT visit 8—episode 5 (minutes 20–50). While playing, the volume will be standardized, and captions will be on for all participants.

#### Therapy sessions

Fifty-minute CBT sessions will occur weekly for 8 weeks with content based on a standardized CBT manual developed by the South Central MIRECC [20]. Each session will be recorded by the Lyssn platform (Lyssn, Seattle, WA, USA), providing the opportunity for ongoing supervision and fidelity checks [29]. Treatment will begin with introducing the CBT model, integrating the patient’s lived experiences to facilitate buy-in and build positive expectations for treatment outcomes. Therapists will provide the rationale for CBT and overarching goals of treatment and establish the role of out-of-session assignments of behavioral homework with verbal commitment being elicited. During the first session, the Columbia Suicide Severity Rating Scale (C-SSRS), which was completed during the intake visit, will be reviewed with the therapist. The therapist will help to create a collaborative safety plan for repeated reference in each session.

Briefly, the standardized weekly CBT sessions will look as follows: session 1 will focus on the above items and conclude with establishing at least one behavioral goal to be completed before session 2. In session 2, thoughts will be reviewed as antecedents of behavior and maladaptive thoughts and the Dysfunctional Thought Record (DTR) will be introduced. An initial DTR will be assigned for homework. In session 3, the DTR from the previous week will be evaluated and further explored. In addition, “Hot Thoughts” and cognitive distortions will be introduced. In session 4, skills for challenging maladaptive thoughts will be introduced and practiced. In addition, behavioral activation will be introduced and implemented in and between sessions via SMART goals. In session 5, participants will explore behavioral activation in more detail, discussing the typical barriers and making a plan to implement new activities. In session 6, the participant will learn about problem-solving skills and practice a problem-solving model with a specific problem they identify. Session 7 will provide an introduction to relaxation practices, including diaphragmatic breathing and progressive muscle relaxation. In addition, the participant will start to develop a maintenance plan for once therapy is concluded. In the final session, treatment progress will be reviewed by the participant and the therapist, noting the central treatment processes. Continued practice of the learned skills will be emphasized as a mechanism for relapse prevention.

Immediately after each therapy session, questionnaires will again be administered. In sessions 1, 4, and 8, participants will have a blood sample taken. Additionally, after sessions 4 and 8, participants will be asked to re-wear the activPAL accelerometer for 7 days and return during their next visit (session 5 and post-therapy visit, respectively).

### Criteria for discontinuing or modifying allocated interventions {11b}

Due to safety concerns, participants will discontinue participation in the study if the participant is deemed to require higher-level care via an occurrence of any of the following: (1) the participant is hospitalized overnight for suicidality, (2) the participant is rated as a 5 (“Active Suicidal Ideation with Specific Plan and Intent”) on the suicidality portion of the C-SSRS at any point, (3) law enforcement is called and transports the participant to emergency services, or (4) the participant attempts suicide at any point during the study. Language regarding this preclusion will be included in the informed consent document for participants to review before enrolling in the study. Any hospitalization or any participant exiting the study for the above reasons will be immediately reported to the Data and Safety Monitoring Board (DSMB) to review and provide study recommendations if needed.

Medication-related issues will be minimized with the inclusion criterion that participants are on a stable mental health medication regimen (if they are taking any mental health medications) for the past 8 weeks and willing to maintain treatments throughout the study. Change or the start of new medication during study enrollment will be discouraged but will not preclude continued participation in the intervention. Any medication changes will be recorded.

### Strategies to improve adherence to interventions {11c}

Adherence to study visits will be facilitated by (1) using a unique study identity (i.e., “the CBT + study”), with all participant materials using a lab-specific logo, specific colors, and font; (2) having the study team well organized with substantial training on participant interactions, including protocols of standard responses to possible participant questions and interactions; (3) having thorough informed consent processes (described above); (4) offering flexible scheduling (e.g., morning, daytime, evening appointments); (5) having a consistent person involved in scheduling and study team communications (e.g., project coordinator); (6) sending automatic email reminders coupled with regular reminder texts and phone calls; and (7) making multiple attempts to recontact and reschedule when needed.

The promotion of participant retention will be done in several ways including automated email reminders for each visit, visit reminder cards, and personalized data (i.e., ActivPAL reports) given at follow-up visits.

### Relevant concomitant care permitted or prohibited during the trial {11d}

Inclusion criteria (i.e., either not engaging in any mental health treatment [e.g., medication, psychological, or behavioral] or on a stable mental health treatment regimen and willing to maintain it for the length of the intervention) will be used to minimize any impact of concomitant care. If participants report engaging in new treatment during the intervention period, the study team will document the occurrence but not terminate study participation. This is designed to allow participants into the trial who would benefit from starting a new treatment and minimize the potential of extraneous treatment changes to confound the study results.

### Provisions for post-trial care {30}

All participants will receive a mental health resource sheet providing a list of crisis resources if they need immediate help during and following study enrollment. Regardless of risk, during the final CBT session, the therapist will provide all participants with information about local mental health clinics. After the post-therapy visit when primary outcomes and clinical endpoints are collected, participants can engage in any additional treatment without informing the study team. During the post-therapy visit, if suicide-related items are endorsed via the SCID or C-SSRS, the safety plan will be referenced and updated as needed. The clinical interviewer completing the post-therapy visit will assist the participant in making connections with appropriate mental health services, local emergency services, or continuing outpatient care as interested and as needed.

### Outcomes {12}

All data will be recorded electronically in REDCap [33] and secured on a password-protected drive. Descriptions of all primary and secondary outcomes are described below, with a schedule of when each is administered presented in Table 1.

**Table 1:**
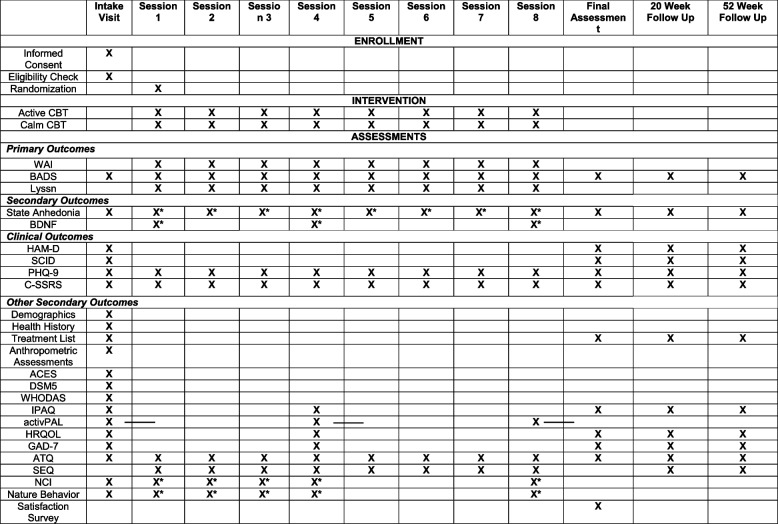
Timing of assessments across the 8-week intervention and 52-week study involvement

Anthropometric assessments = height, weight, heart rate, blood pressure

activPAL issued at the visit and worn for 7 days before being returned at the next visit or provided 7 days before Final Assessment (indicated by x ——)

^a^Measures taken 3 times (pre-condition, post-condition, and after therapy)

#### Primary outcomes: target CBT mechanisms

The primary outcomes of this trial are participant-rated working alliance and change in behavioral activation each averaged across all eight sessions. These CBT mechanisms were chosen due to their well-established relationship with CBT treatment outcomes [4, 48, 61, 64] to best capture process effects during (working alliance) and between sessions (behavioral activation) to predict antidepressant outcomes due to the course of therapy. Working alliance is an important predictor of success across all therapy types (i.e., a “common” factor). In contrast, behavioral activation represents a specific factor focused on during CBT (i.e., a “specific” factor to CBT). Therefore, these two targets will assist in determining if exercise priming may be useful in general across therapy types (e.g., it leads to higher working alliance) or, more specifically, useful in CBT (e.g., it leads to higher behavioral activation).

##### Working Alliance Inventory—Short Revised (WAI-SR)

At the end of each CBT session, participants will rate their working alliance with their therapist via the WAI-SR, a 12-item assessment using a Likert-style rating scale ranging from 1 (seldom) to 5 (always). The sum of this measure will be used in the primary outcome, with higher scores indicating greater therapeutic alliance [34]. Comparisons between groups will be made using the weekly (weeks 1–8) and assessment time (i.e., intake visit and post-therapy visit) group averages. The WAI-SR is highly correlated with the full WAI, with strong subscale (*α* = 0.85–0.90) and total score (*α* = 0.91) psychometric properties [34].

##### Behavioral Activation for Depression Scale (BADS)

Behavioral activation will be assessed prior to completing the preparatory condition via the Behavioral Activation for Depression Scale (BADS; [37]) consisting of 25 questions inquiring about activation, avoidance/rumination, work/school impairment, and social impairment over the last week. Some items are reverse scored and then summed for a total score, with higher ratings indicating greater behavioral activation [40]. Similarly, comparisons between groups will be made using the weekly (weeks 1–8) and assessment time (i.e., intake visit and post-therapy visit) group averages. This questionnaire has demonstrated acceptable overall (*α* = 0.79) and subscale (all *α* > 0.78) internal consistency. Construct validity of this scale has been established via correlations with Beck Depression Inventory (*r* =  − 0.67), Automatic Thoughts Questionnaire (*r* =  − 0.62), Acceptance and Action Questionnaire (*r* =  − 0.51), Cognitive Behavioral Avoidance Scale (*r* =  − 0.37), and Response Styles Questionnaire (*r* =  − 0.56; [37]).

##### Sensitivity of objective machine learning-based markers of CBT (Lyssn)

Advances in machine learning and artificial intelligence have transformed computers’ abilities to create and respond to natural language. For example, our team members and colleagues at Lyssn previously trained a machine learning model to predict working alliance from therapy recordings [29]. Lyssn team members led the foundational research establishing that machine learning-based evaluation of psychotherapy quality is possible [1]. The Lyssn platform employs algorithms that utilize speech features to identify both session-level (e.g., how collaborative was the therapist in this session?) and per-utterance (e.g., open questions, affirmations, confrontations within talk-turns) objective coding for a variety of CBT, motivational interviewing, and other metrics. This previously developed tool will be used in the present project to provide objective assessments for each therapy session (e.g., by providing a collaboration and empathy score) to complement the WAI-SR participant ratings. It will be used to assess the ability of objective/algorithm-derived methods to identify between-group differences in therapy in CBT sessions 1–8 to complement any differences identified in the self-report primary outcome measures.

#### Secondary outcomes: exercise priming mechanisms

Secondary outcomes that explore exercise priming mechanisms are serum brain-derived neurotrophic factor (BDNF) and state anhedonia.

##### Brain-derived neurotrophic factor (BDNF)

Given that preliminary data suggests that 30 min of aerobic exercise increases serum BDNF [51], blood draws will be completed before the condition, after the condition but before therapy, and after therapy (3 time points,PRE, MID, POST) at sessions 1, 4, and 8. Average changes in BDNF levels from the pre-therapy condition (i.e., pre-condition to post-condition) during sessions 1, 4, and 8 will be used to evaluate group differences in this outcome. The serum content of BDNF will be assessed via enzyme-linked immunosorbent assay (R&D Systems, Minneapolis, MN; as done previously by the research team [51]). The serum will be stored at − 80 °C until analysis before batch processing according to the manufacturer’s instructions.

##### State anhedonia

As our pilot data suggests, 30 min of moderate-intensity aerobic exercise decreases state anhedonia [52, 53], acute changes in state anhedonia will be assessed via an anhedonia visual analog scale (VAS) and the Dimensional Anhedonia Rating Scale (DARS [58]). Using the VAS, participants respond to the prompt “Drag the slider to rate how you are feeling right now,” utilizing a scale from 0 (“No pleasure at all”) to 100 (“Extreme pleasure”). The DARS is a 17-item self-report survey that assesses state anhedonia across four domains: hobbies, food/drink, social activities, and sensory experiences. At the intake visit, participants will list two activities within each domain that they consider their favorite and then respond to questions regarding their current interest in and enjoyment of those activities with ratings on a 5-point Likert scale from “Not at all” to “Very much” with higher scores representing less anhedonia [58]. Participants will be asked to respond to how they are feeling “RIGHT NOW” to evaluate their in-the-moment feelings. Favorite items recorded at the intake visit will then be used as the prompt for each subsequent administration of the instrument. To determine changes in anhedonia, assessments will be completed at the intake, post-therapy, and 20- and 52-week follow-up visits, as well as pre-therapy condition, post-therapy condition, and after all CBT visits. The primary comparison between groups will be made using average scores from each session time (i.e., pre-therapy condition, post-therapy condition, post-therapy) point during each CBT + visit. This questionnaire has demonstrated high internal consistency (*α* = 0.96) for the total score and across all domains (*α*s > 0.88) in individuals with MDD, as well as acceptable convergent validity (*r* = 0.79 with Snaith-Hamilton Pleasure Scale; [58]).

#### Clinical outcomes

##### Depressive symptom severity

As depression severity ratings are the major clinical outcome of interest in this trial for adults with MDD, depression symptom severity will be assessed using the gold-standard GRID-Hamilton Rating Scale for Depression (GRID-HAMD) [30, 74]. The GRID-HAMD will be completed at all assessment visits (i.e., intake, post-therapy, 20-week follow-up, 52-week follow-up). It is a 17-item clinician-completed questionnaire used to rate depressive symptom severity by probing mood, feelings of guilt, suicidal ideation, insomnia, agitation or retardation, anxiety, weight loss, and somatic symptoms [31]. Higher scores reflect greater symptom severity. GRID-HAMD scoring is categorized as no depression (0–7), mild depression (8–16), moderate depression (17–23), and severe depression (≥ 24). Changes in total scores from the intake visit to post-therapy visit will be used to examine group differences, with changes at the follow-up visits used to explore longer-term effects of the intervention. Interviews will be conducted by advanced trained interviewers who are blinded to treatment assignment and have undergone systematic training in the use and scoring of this instrument. The original HAMD (without GRID scoring) has demonstrated acceptable reliability (internal consistency of *α* = 0.46–0.97; inter-rater reliability of 0.82 to 0.98; and test–retest reliability of 0.81 to 0.98) and validity (highly correlated [*r* = 0.89] with physician global ratings of depression) [8, 39]. Inter-rater reliability has been found to be further improved by using GRID scoring [67].

To track weekly changes in self-reported depression severity throughout the study, depression severity will also be self-reported via the Patient Health Questionnaire-9 (PHQ-9) at each study visit. This is a 9-question self-report instrument used to assess presence and severity of depressive symptoms, with scores categorized as minimal (0–4), mild (5–9), moderate (10–14), moderately severe (15–19), and severe (20–27) depression [41]. Changes in total scores between the intake visit and post-therapy visit will be used to examine group differences. This questionnaire has demonstrated excellent internal reliability (*α* = 0.89; [41]).

##### Clinical diagnosis of depression

The Structured Clinical Interview for DSM-5 Disorders (SCID [28]) will be used to identify any exclusionary psychological diagnoses (e.g., psychosis), confirm a clinical diagnosis of MDD, and to track changes in meeting diagnostic criteria. Relevant SCID sections (i.e., mood episodes, psychotic and associated symptoms, substance use disorders, and anxiety disorders) will be administered at each assessment visit by a trained interviewer at each assessment visit. Additional diagnoses from sections not administered (e.g., ADHD, PTSD) will be systematically self-reported via questions about mental health history and previous diagnoses at the intake visit. The SCID is a semi-structured interview for making diagnoses according to the diagnostic criteria published in the American Psychiatric Association’s Diagnostic and Statistical Manual for Mental Disorders (DSM-5,2013). Interviews for the SCID will be conducted by trained interviewers (e.g., counseling psychology doctoral students) blinded to treatment assignment who have undergone systematic training in using the SCID for determining clinical diagnoses. That training will include four steps. First, interviewers will complete the official SCID Didactic Training Course Video. Second, interviewers will watch official training interviews of roleplayed clients with different diagnoses and will complete a SCID while watching. Third, the interviewers will meet with Dr. Wade to discuss the training, practice key interview skills, and review their SCID ratings against the official keys provided by the SCID developers. Finally, interviewers will meet with Dr. Thomas, who will roleplay a depressed client and then assess the fidelity of the interviewer’s ratings. Once interviewers demonstrate competency to accurately assess in a live roleplay situation, they will be cleared to start assessing participants. Interviewers will then meet weekly with Dr. Wade to supervise the assessments, discuss participant cases, and watch videotapes of interviews as needed. Rates of meeting MDD diagnostic criteria at intake and post-therapy will be the primary time points used to compare remission rates between groups.

##### Suicide risk

As suicidal ideation is a potential safety risk for enrolled participants, suicide risk will be monitored throughout the duration of the study. Suicide risk will be assessed via the Colombia Suicide Severity Rating Scale (C-SSRS) administered by the clinical interviewer (assessment visits) or therapist (CBT sessions) during all study visits. All clinical interviewers and therapists will complete the C-SSRS Training for Clinical Practice and obtain certification before administering the assessment. Implementation of the study’s suicide safety plan is based on severity level using this measure.

#### Other measures

##### Participant characteristics

To characterize the sample, during the intake visit participants will report demographic information, including age, gender, sex, race, ethnicity, marital status, education level, employment status, and income. They will also be asked to list and describe their current mental health treatments (e.g., therapy, medication), in addition to listing their history of and current health condition (e.g., cancer, heart attack, diabetes). Averages and percentages of these items will be used to characterize the sample.

##### Physical activity and sedentary time

To assess physical activity, participants will self-report physical activity via the International Physical Activity Questionnaire-Short Form (IPAQ-SF) [16]. The IPAQ-SF will be administered verbally at intake, CBT session 4, post-therapy, and both follow-up visits. Changes in moderate-to-vigorous physical activity, walking time, and sedentary time at each time point will explore group differences. This questionnaire has demonstrated high reliability (*α* < 0.80; [16]).

To support behavioral activation and physical activity self-reports, physical activity and sedentary time will be monitor assessed via an accelerometer (ActivPAL4, PAL Technologies Ltd., Scotland, UK). Participants will be provided instructions and asked to wear the activPAL accelerometer for 7 days (24 h/day) at the intake visit, CBT session 4, and CBT session 8. The activPAL accelerometer assesses activity through monitoring time spent standing, sitting, laying, and stepping, as well as sleeping. Changes in those behaviors at each time point will be used to evaluate group differences.

##### DSM-5 Level 1 Cross-Cutting symptoms measure

The DSM-5 Level 1 Cross-Cutting Symptoms Measure is a self-rated measure consisting of 23 questions used to assess mental health domains that aid in psychiatric diagnosis [47]. There are 13 domains assessed with this measure: depression, anger, mania, anxiety, somatic symptoms, suicidal ideation, psychosis, sleep problems, memory, repetitive thoughts and behaviors, dissociations, personality functioning, and substance use over the previous 2-week period. This measure will be completed during the intake visit only, and averages will be used to characterize the sample.

##### World Health Organization disability assessment schedule

The World Health Organization Disability Assessment Schedule (WHODAS) is a self-rated measure consisting of 12 questions to assess difficulties arising due to health conditions, including disease/illness or other long-lasting health concerns, injuries, mental health problems, and substance use. This questionnaire assesses the difficulty of performing everyday life tasks over the past 30 days. Response choices range from “None” (1) up to “Extreme or cannot do” (5), with higher scores indicating greater levels of difficulty due to a health condition. Studies have found this measure to have high validity (*α* = 0.81–0.96) and correlations with other disability scales [60]. The WHODAS will be completed at the intake visit only, with averages used to characterize the sample.

##### Quality of life

The SF-36 Health Survey will be used to examine the effects of the intervention on quality of life. The SF-36 consists of 36 questions used to assess health status using a multi-item scale assessing eight health sections: (1) limitations in physical activities because of health problems, (2) limitations in social activities because of physical or emotional problems, (3) limitations in usual role activities because of physical health problems, (4) bodily pain, (5) general mental health, and (6) limitations in usual role activities because of emotional problems [72]. Subscale scores will be calculated using standardized scoring procedures. The SF-36 will be administered during all assessment visits and CBT + session 4, with scores on each sub-scale between time points used to examine differences in quality of life between groups.

##### Anxiety

As anxiety has high comorbidity with depression, symptoms of anxiety will be assessed via the Generalized Anxiety Disorder-7 (GAD-7) [63]. The GAD-7 is a self-administered 7-item questionnaire where participants provide ratings (0 = not at all, 1 = several days, 2 = more than half the days, 3 = nearly every day) to seven anxiety-related questions using the prompt “over the last 2 weeks, how often have you been bothered by the following problems?.” The GAD-7 is scored as the sum of all responses, with scores of 0–5, 6–10, and 11–15 being the ranges for mild, moderate, and severe anxiety, respectively. This questionnaire has demonstrated excellent internal consistency (*α* = 0.92) and good test–retest reliability (intraclass correlation = 0.83). Convergent validity was determined via correlations with the Beck Anxiety Inventory (*r* = 0.72) and the anxiety subscale of the Symptom Checklist-90 (*r* = 0.74; [63]). The GAD-7 will be administered during all assessment visits and CBT + session 4, with total scores at each time point used to examine differences in anxiety between groups.

##### Session effectiveness

Given that CBT focuses on reducing negative automatic thoughts, the Automatic Thoughts Questionnaire (ATQ; measuring CBT-related cognitions [35]) will be administered at each study visit (i.e., CBT session and assessment visits). The ATQ is a 30-item self-reported instrument that measures the frequency of automatic negative statements about the self. Participants rate items on the frequency of occurrence from 1 (“not at all”) to 5 (“all the time”). The ATQ is scored as the sum of all 30 items, which can be separated into four aspects of automatic thoughts (i.e., personal maladjustment and desire for change, negative self-concepts and negative expectations, low self-esteem, and helplessness). A high total score indicates a higher level of automatic negative self-statements. The ATQ total score at the intake visit and the post-therapy visit will be the time points and metrics of interest to explore group differences for this outcome. The ATQ has previously shown acceptable internal reliability and concurrent validity [35].

Additionally, to evaluate the participants’ perspective of the quality of the session itself, the Session Evaluation Questionnaire (SEQ) [65] will be administered at the end of each CBT session as well as the two follow-up visits. The SEQ includes 21 items in a 7-point bipolar adjective format that queries how the participant feels about each CBT session, with subscales of session depth, smoothness, positivity, and arousal. The stems of “This session was” and “Right now I feel” with different scale anchors (e.g., “bad” or 1 to “good” or 7; “happy” or 1 to “sad” or 7) will be used to calculate subscales using standardized scoring procedures. Average scores on the SEQ from each CBT session will be used to examine group differences in perceived session quality. Previous studies have found adequate to good internal consistency (*α* = 0.77–0.92) for the SEQ [65].

##### Adverse childhood experiences

As high levels of childhood adverse events are related to adult depression [27], the occurrence of adverse childhood experiences will be assessed at intake assessments via the Adverse Childhood Experience Survey (ACES [17]). The ACES consists of 16 questions pertaining to the respondents’ first 18 years of life. Participants respond with yes or no regarding the occurrence of the adverse event. Participants are scored as exposed for each event they are exposed to, with scores ranging from 0 (unexposed) to 7 (exposed to all categories). Total scores from baseline will be used to examine the influence of childhood experiences on treatment effects of the intervention.

##### Intervention satisfaction

As Acceptability of Implementation Measure (AIM), Implementation Appropriateness Measure (IAM), and Feasibility of Intervention Measure (FIM) are indicators of implementation outcomes [73], each will be self-administered at the post-therapy, 20-week, and 52-week follow-up visits. Four items will be used to assess each outcome with response choices ranging from 1 (completely disagree) to 5 (completely agree), with higher total scores indicating higher satisfaction with the treatment provided. Average scores from the post-therapy visit will be used as the primary time point to explore intervention satisfaction for each group. These measures have previously demonstrated high validity, reliability, and response to change [73].

##### Nature connectedness

Nature connectedness is positively correlated with happiness and subjective well-being [57], so to monitor the effects of viewing the nature documentary each week, the Nature Connectedness Index (NCI; [57]) will be administered at each assessment visit, as well as pre-condition, post-condition, and post-therapy on CBT sessions 1, 4, and 8. Scores on this questionnaire range from 0 to 100, with high scores reflecting greater feelings of connection to nature, with Richardson et al. [57] showing the measure has been a reliable and valid tool for exploring this construct. Average changes in nature connectedness from pre/post-condition and post-therapy during CBT sessions 1, 4, and 8 will be used to evaluate group differences. Additionally, the Nature Based-PA questionnaire will be used to assess participants’ accessibility to nature-based physical activity to help in understanding how the intervention relates to nature connectedness. This will be assessed by asking two yes/no questions: “Do you currently have the option to be active in or around natural environments?” and “In the past 7 days, have you participated in any nature-based physical activity?.” If yes to both questions, participants will indicate the number of days per week they engaged in nature-based physical activity.

#### Protocol adherence measures

##### Video stimulus neutrality

A Nature Video Survey will be assessed following each pre-therapy condition to monitor emotional responses to viewing the animal documentary. The goal of showing the video is to create an emotionally neutral stimulus for each group that does not elicit overly negative or positive feelings. Four bipolar questions will inquire about how emotionally negative/positive (negative/positive), arousing or exciting (calm/exciting), entertaining (very unentertaining/very entertaining), and how the video compares to the previous video (much less entertaining/much more entertaining) reported on a 1–10 visual analog scale. Average scores from each CBT session will be used to examine stimulus neutrality by group.

##### Perceived condition effort

Active and calm condition exertion intensity will be monitored via heart rate and the rating of perceived exertion (RPE; [10]) scale during the warmup and every 5 min during the 30-min condition. Participants in the active condition will be encouraged to exercise at a moderate intensity (or “somewhat hard,” a 13 on the scale), while participants in the calm condition will be encouraged to rest comfortably (anticipating RPE ratings of 6 or “No exertion at all”). Standard RPE instructions will be provided during the first pre-therapy condition, with abbreviated instructions before all subsequent sessions. Average RPE ratings during each condition at each CBT session will be used to report perceived effort for each group.

## Visits and study flow

### Participant timeline {13}

**Fig. 1:**
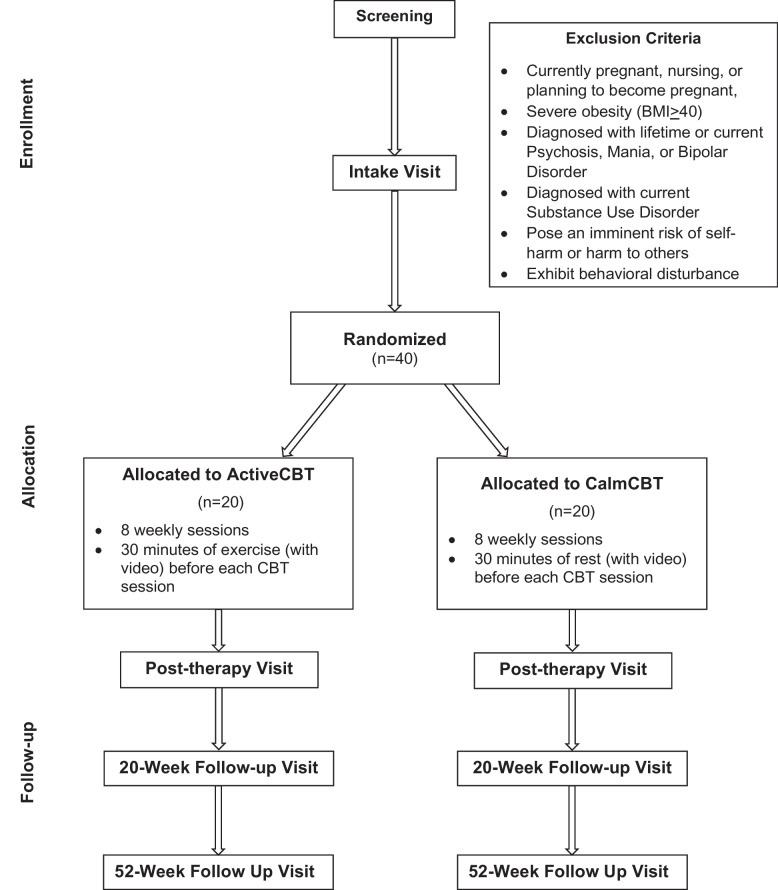
CONSORT diagram of study design of the CBT + trial

The CONSORT diagram showing an overview of the study flow is displayed in Fig. 1.

#### Screening

All recruitment materials (e.g., emails, flyers) will include a link or QR code that directs interested participants to complete a pre-screening electronic survey on REDCap to self-report basic eligibility criteria including completing the Patient Health Questionnaire-2 (PHQ-2). Individuals who screen as potentially eligible (i.e., meet age criteria and have a PHQ-2 ≥ 3, indicative of a high likelihood of having current depression [6]) will be contacted for a follow-up phone screening survey. During this call, participants will learn more about the study, be able to ask any questions, and verify information from the online screener. The phone screening will ask additional mental health questions, such as the PHQ-8, to better understand current depressive symptoms and will collect PAR-Q information to verify the safety of exercise. Individuals who appear potentially eligible from the phone screening survey (i.e., meeting all criteria from online screening, safe to participate in exercise from PAR-Q, and PHQ-8 ≥ 10; [42]) will schedule an intake visit.

#### Intake visit (~ 3 h)

During the intake visit, potential participants will first complete the informed consent process (detailed above). Following, height and weight will be measured, and BMI exclusion criteria will be verified. Next, the participant will complete a mental health clinical interview, administered by a trained clinical interviewer. The clinical interviewer will determine if the participant meets CBT-naïve criteria and inquire about current or previous history of additional, non-exclusionary mental health diagnoses, which will be used to help characterize the sample (i.e., diagnoses systematically self-reported to clinical interviewers). Next, the following sections of the SCID will be completed: (1) nonpatient overview, used to build rapport with the clinical interviewer, (2) mood episodes, to confirm a current MDD diagnosis and screen for current or lifetime mania, (3) psychotic and associated symptoms, to screen for current or lifetime diagnosis of psychosis or bipolar disorder, (4) substance use disorders, to screen for current substance use disorder, and (5) anxiety disorders, to document co-occurring anxiety disorders due to high comorbidity. If the participant is diagnosed with current MDD and no exclusionary criteria from the SCID, the clinical interviewer will administer the Hamilton Rating Scale for Depression-17 (GRID-HAMD) to determine current depressive symptom severity and the Columbia Suicide Severity Rating Scale (C-SSRS) to assess suicide risk. Participants will be excluded if they do not meet the severity threshold (HAMD ≥ 8) or if they have active suicidal ideation with a specific plan and intent based on the C-SSRS (i.e., 5 out of 5 on the suicidality screen). Regardless of suicide risk or eligibility, clinical interviewers will next follow the suicide risk assessment and safety planning and collaboratively create a safety plan with the participant.

Participants who meet all inclusion criteria will then complete a demographic questionnaire and health history form and list their medications. Next, the research staff will verbally administer the International Physical Activity Questionnaire-Short Form (IPAQ-SF). Participants will then complete questionnaires including the Patient Health Questionnaire (PHQ-9), anhedonia visual analog scale (VAS), Dimensional Anhedonia Rating Scale (DARS), Nature Connectedness Index (NCI), Nature Behavior, Behavioral Activation for Depression Scale (BADS), Automatic Thoughts Questionnaire (ATQ), General Anxiety Disorder-7 (GAD-7), and Short-Form Health Survey (SF-36) with all questionnaires being completed electronically via REDCap. Next, baseline blood pressure and heart rate will be taken. Finally, participants will be given instructions and asked to wear an activPAL accelerometer for 24 h/day for the following 7 days and schedule their weekly CBT sessions.

#### Weekly CBT sessions (~ 2 h)

Across the 8-week intervention, participants will arrive ~ 50 min before the start of their CBT session. Each session will begin by completing anhedonia VAS and DARS, PHQ-9, BADS, and ATQ questionnaires. On CBT sessions 1, 4, and 8, a blood sample will be collected following these questionnaires. Additionally, the SF-36, IPAQ-SF (verbally), NCI, and GAD-7 will be completed in CBT session 4. Participants will then complete their pre-therapy condition (detailed above). Following their active or calm condition, participants will complete the anhedonia VAS, DARS, and video survey. During sessions 1, 4, and 8, another blood sample will be taken following the questionnaires. Ten minutes after the active and calm condition ends, participants will be escorted to a private room to meet with a therapist to begin a 50-min individual CBT session. Immediately after each therapy session, the SEQ, WAI, anhedonia VAS, DARS, and NCI will be completed. In sessions 1, 4, and 8, participants will have a third blood sample taken following the questionnaire completion. Additionally, after sessions 4 and 8, participants will re-wear the activPAL accelerometer to return at their next visit.

#### Post-therapy, 20-week, and 52-week follow-up visits

Participants will return roughly 1 week after their final therapy session (i.e., session 8) for a post-therapy visit. During the post-therapy visit, a mental health clinical interview will be completed that is identical to that conducted at intake (i.e., SCID, GRID-HAMD, and C-SSRS). Additional final assessment measures include IPAQ-SF, SF-36, NCI, Nature Behavior, PHQ-9, GAD-7, BADS, ATQ, anhedonia VAS, DARS, Satisfaction Survey, height, weight, blood pressure, and heart rate. Participants will also return for follow-up visits at 20 weeks (roughly 12 weeks after the end of therapy) and at 52 weeks total to determine potential longer-term effects, with both visits identical in procedures to the post-therapy visit.

### Sample size {14}

R and R Studio were used to determine sample size and will be used to complete all analyses. The sample size of 40 was determined to ensure a high probability for achieving the pre-set criteria to move forward to a larger trial for mechanistic target engagement under the expected moderate effects of Cohen’s *d* ≥ 0.50 and a low probability for achieving the “Go” criteria when Cohen’s *d* = 0 for the primary endpoints of working alliance and behavioral activation. For each endpoint, Cohen’s *d* reflects the difference in pooled standard deviation units between ActiveCBT and CalmCBT for the expected outcome value averaged across CBT sessions 1 through 8. The threshold for a recommendation of progression to a larger trial of this approach is finding a between-group difference favoring ActiveCBT in either WAI or BADS of Cohen’s *d* ≥ 0.55 or both at *d* ≥ 0.35. These analyses conservatively assume 32 of the 40 participants provide complete data (80% retention), though we expect to retain more than 32 participants, given > 90% retention in the pilot trial [52, 53], and we will incorporate partial endpoint data in these analyses from participants who withdraw (i.e., all available data until participant withdrawal). When true target engagement is absent on both endpoints (*d* = 0), the Go criteria are unlikely to be achieved (2% chance or lower), and conversely, when true target engagement is moderate on both endpoints (*d* ≥ 0.50) or strong (*d* ≥ 0.75) on one endpoint, the Go criteria are very likely to be achieved (74% chance or higher). If “Go” criteria are met, a subsequent trial would be conducted to confirm greater engagement of these working mechanisms of CBT with sufficient power to detect a clinically meaningful between-group difference on the endpoint of depression severity (HAM-D).

### Recruitment {15}

Forty adults will be recruited from Ames, IA, and the surrounding area. Participants will primarily be recruited via referrals from local community hospitals, local clinics, and outpatient mental health treatment centers. This will be supplemented by mass emails to individuals who previously expressed interest in the study, the university community, and large local employers. In addition, community flyers and social media posts will be used.

## Assignment of interventions: allocation and masking

### Randomization—sequence generation and implementation {16a, 16c}

Randomization will be severity-stratified (mild vs moderate to severe based on GRID-HAMD scores) using a 1:1 permuted block randomization. Stratification by severity was chosen to ensure a similar distribution of severity within each group, as baseline severity is a consistent predictor of post-treatment depression [23, 24]. The study statistician set up the randomization module on REDCap and uploaded the allocation table, based on depression severity (i.e., severity-stratified). Senior research staff will enter required fields on REDCap (i.e., symptom severity level) at the beginning of the first CBT session when all baseline data is collected (i.e., activPAL), which will automatically generate the group assignment.

### Masking—randomization, study team, and unblinding procedures {16b, 17a, 17b}

The principal investigator, statistician, and clinical interviewers will be masked to participant assignment until the trial is complete and all pre-specified analyses have been completed. Masking participants to treatment group assignment is not possible. However, efforts will be made to mask therapists to participant assignment to maintain treatment similarity and decrease treatment allegiance. As such, a 10-min recovery time frame after the active/calm condition and before the therapy session will be used to limit incidental group membership disclosure due to after-effects of the condition (e.g., increased heart rate or breathing rate from the active condition). In addition, to systematically monitor incidental group disclosure, therapists will record if any conversational disclosure of group assignment (i.e., active or calm) happens, and analyses will be performed both with and without sessions in which group membership was disclosed or known (e.g., if the group is disclosed by the participant in week 6, therefore it is known by the therapist in weeks 6, 7, and 8). Finally, therapists and clinical interviewers will complete a formal assessment after completion of the last therapy session or last clinical interview, which asks if they discussed group assignment at any point with the participant and to guess what group they thought the participant was allocated to.

### Data collection, management, and confidentiality {18a, 18b, 19, 27}

REDCap will be used as the primary data collection platform. Data from study visits (e.g., informed consent signatures, questionnaires, and raw data) will be directly entered on REDCap during each study visit. Data collected on other software (e.g., activPAL, Lyssn) will be imported to REDCap following study visits. All possible REDCap data validations (e.g., numerical ranges, formatting) will be set to promote data quality. Data will be reviewed weekly for missingness and accuracy using the REDCap Data Quality feature and weekly reports generated by the team statistician. Cloud storage will also be used as a backup data storage platform.

Confidentiality will be ensured by using a unique study identifier (e.g., CBT + 603) on all study data. The study team will have one key linking the unique study identifier to personally identifying information, which will be stored on a password-protected platform, only accessible to senior research.

Participant retention will be promoted by using a unique study identity, with all participant materials using a lab-specific logo, specific colors, and font. In addition, during the informed consent process, to build trust and demonstrate transparency, pictures and a description of the study team will be presented. Easy-to-understand visual representations of study procedures will be used to better describe what enrollment entails and the importance of high adherence. Next, participants will be given a tour of the facility with the ability to ask questions. Lastly, during study enrollment participants will be offered flexible scheduling for appointments and convenient access to the testing building (e.g., parking, greeting at the door). If participants miss a visit, multiple attempts to recontact and reschedule will be made (e.g., email, text, and call).

### Plans for collection, laboratory evaluation, and storage of biological specimens for genetic or molecular analysis in this trial/future use {33}

As described above, serum samples will be used for analysis of BDNF content. Samples will be aliquoted and stored in a − 80°C freezer until analysis will occur via ELISA. Additional samples may be stored for future use as described in the consent document with a specific focus on biomarkers related to exercise, depression, or treatment.

### Statistical methods {20a, 20b, 20c, 31c}

#### Plans for assessment and collection of outcomes

Study outcomes will be collected longitudinally and analyzed using generalized estimating equations (GEEs). In general, the mean value under each condition, and the difference in mean value between conditions, will be estimated at each ascertainment time point and visualized graphically. All statistical significance testing will be carried out at the two-sided 0.05 level according to the intention to treat principle, including all participants that are randomized. All data collected before a participant’s withdrawal will be incorporated into analyses when feasible. Analyses will performed using R version 4.3.1 or newer [56]. Participant-level data will be shared with the NIMH Data Archive, as required by NIMH-funded research.

The study’s primary aim is to determine the engagement of target CBT mechanisms by exercise priming in the active condition above and beyond that which is engaged in by the calm condition. Therefore, the primary aim will be evaluated by group comparisons of the average and assessment time point-specific observed effects of working alliance and behavioral activation. A GEE will be used to evaluate temporal heterogeneity in the effect of condition group on WAI over the eight CBT sessions. Change in BADS relative to visit one (which will be an assessment of the week prior to the first CBT session) will be analyzed similarly. The estimated mean WAI and change in BADS at each session will be averaged and standardized by the pooled standard deviation with the difference between groups reflecting Cohen’s *d.* As a sensitivity analysis, we will adjust for HAM-D at baseline and additional predictive patient characteristics collected at baseline with observed imbalances across groups (e.g., *p* < 0.2) as well as for therapist.

The link between psychological and neuroplastic exercise priming to CBT mechanisms will be evaluated using repeated measures correlation analyses of change in pre- versus post-exercise BDNF and DARS, and their mediating effects on WAI and change in BADS. GEEs will be implemented to facilitate verifying and evaluating the extent to which moderate exercise causes acute effects on BDNF and DARS. Mediation analyses will be carried out to estimate the direct and indirect effect of the active condition on WAI and change in BADS. As exploratory analyses, we will evaluate condition effects similarly using GEEs on Lyssn session metrics (collaboration and empathy) and secondary clinical outcome measures.

### Interim analyses {21b}

There are no plans for interim analyses for the present trial.

### Oversight and monitoring {5d, 21a}

A Data and Safety Monitoring Board (DSMB) has been formed to independently monitor participant safety, data quality, and the progress of the trial. The DSMB includes a clinical psychologist (chair), a clinical psychologist with expertise in depression (DSMB member), and a clinical trials biostatistician (DSMB member). The DSMB will meet every 6 months throughout participant participation and complete interim analyses as necessary. The local IRB will also closely monitor study procedures and ethics, meeting biweekly to discuss protocol modifications and reportable events.

### Adverse event reporting and harms, auditing, and communication plans {22, 23, 25}

Reportable events will be systematically documented by the research staff at all study visits during the intervention period. After each intervention study visit, research staff will mark “Yes” or “No” based on whether a reportable event was disclosed by a participant. If a participant indicates that a reportable event occurred, a senior research staff will interview the participant to gather all information necessary to complete a Reportable Event Monitoring Form. In addition, to proactively monitor events, during the post-therapy visit, participants will respond to the question, “Have you experienced changes in medical conditions since you’ve started this trial that is/are potentially related to your enrollment in the study?” If “Yes” is selected, participants will be asked to detail their changes in medical conditions via free text response. MedDRA will be used for classifying all reportable events, and all serious adverse events that warrant protocol changes will be reported in trial publications.

If modifications are necessary, they will be submitted for review to the IRB before implementation. Important protocol amendments will also be discussed with the study sponsor and DSMB and formally reported in the RPRP and regular reports, respectively. Further, the IRB may perform post-approval monitoring as an annual audit of the trial conduct.

### Dissemination plans {31a}

Due to the potential importance of this study in guiding treatment of depression in adults, we will afford considerable time and effort in disseminating all results. First, this trial is registered on ClinicalTrials.gov and results will be posted within 1 year of study completion. Second, results will be disseminated in a manuscript of primary and secondary outcomes, in addition to presentations at national conferences. Data will also be shared with the NIMH Data Archive (NDA).

## Discussion

This is a phase IIc preliminary testing trial within the ORBIT model for behavioral treatment development [21, 55]. It is designed to understand if exercise priming of CBT can engage mechanistic targets while determining the plausibility of the efficacy of this approach for improving depression treatment. If this trial’s results are favorable, a subsequent efficacy trial will be conducted to determine the efficacy of this exercise priming approach for enhancing CBT’s antidepressant effects. If successful, this and the subsequent trial would help to increase the efficacy of therapy for the treatment of depression.

The purpose of this RCT is to determine if pre-therapy exercise augments or “primes” aspects of CBT for depression. Through pre-therapy preparatory activities (i.e., CalmCBT or ActiveCBT), participants may experience a stronger and more quickly developed working alliance (measured via the Working Alliance Inventory) and enhanced behavioral activation (measured via the Behavioral Activation for Depression Scale) during the standardized course of therapy. With a stronger working alliance with one’s therapist and greater behavioral activation during the 8-week study period, depression would be expected to decrease more quickly over time [14]. This trial’s primary focus is on determining if ActiveCBT engages the working mechanisms of therapy success (alliance and activation) before future trials will evaluate ActiveCBT’s effects on depression treatment efficacy. Secondarily, this trial will assess if the acute changes in anhedonia and BDNF differ between the active and calm preparatory conditions and whether these changes relate to the subsequent therapy session alliance and behavioral activation.

Exploratory Lyssn data will be used in two ways: (1) to assess the fidelity of the CBT being provided and (2) to provide non-self-report measures of the therapy sessions to corroborate the self-report findings potentially. Similar to Lyssn’s corroboration of alliance metrics, the accelerometry measurements of activity will objectively evaluate changes in activity patterns that may support any changes in self-reported behavioral activation. Additional secondary outcomes of interest include group differences in therapy’s effects on automatic thoughts, overall post-therapy effects on depression, suicidality, anxiety, quality of life, and relapse across post-intervention time points. We will include a diversified population (e.g., race, sex, and age) and a strong comparator condition (CalmCBT) that will allow us to make generalizable inferences between conditions.

As with all trials, difficult decision points led to various strengths and weaknesses. While including a strong comparator condition provides an attention- and time-match control condition (nature documentary viewing) to the target intervention (exercise), there is not a no-treatment control condition. This may lead to the calm condition experiencing a greater than expected level of clinical benefit and may also have a greater working alliance and behavioral activation (the target therapy mechanisms in this trial) than a standard therapy condition without any preparatory activity. However, the major benefit of using this strong comparator is that any evidence of benefit beyond the calm condition will likely signal real and potentially meaningful therapy augmentation by the active condition and would provide strong support for continued investigation of the exercise priming approach. Additionally, masking in this trial was difficult as the participants could not be masked to their condition. Every effort has been made to mask the therapists by providing a window of time between the end of the pre-therapy condition (i.e., 10 min), which should minimize and hopefully eliminate the physiological effects of the conditions limiting the ability for the therapist to detect which preparatory condition any given participant was assigned. Further, participants will be discouraged from discussing group assignments in therapy or their clinical interviews, and any group disclosure will be recorded with analyses performed both with and without sessions when group membership was known. Together, this trial has been designed to provide a high bar for moving forward to subsequent trials while rigorously investigating a promising approach to augmenting a frontline treatment for depression.

## Trial status

Protocol version 1.0

Protocol version date: June 27, 2024

Date recruitment began: September 1, 2023

Date recruitment completed: Anticipated September 1, 2024

## Data Availability

Primary analysis of CBT + trial data will be conducted by the trial statistician. Other members of the research team will also have access to data to perform any required analysis as per the publication policy and perform secondary analyses. Deidentified data will also be uploaded to NIMH Data Archive at regular intervals throughout the study duration with complete data uploaded by June 2027.

## Abbreviations

CBT: Cognitive behavioral therapy
MDD: Major depressive disorder
ActiveCBT: Active pre-therapy condition group
CalmCBT: Calm pre-therapy condition group
BDNF: Brain-derived neurotrophic factor
IRB: Institutional Review Board
DSMB: Data and Safety Monitoring Board
ORBIT: Obesity-Related Behavioral Intervention Trials
